# Cannabinoid Effects of Metamizol/Dipyrone: A Possible Second Life in Pediatric Anesthesia for a Vintage Drug

**DOI:** 10.3390/biomedicines14020358

**Published:** 2026-02-04

**Authors:** Alessandro Vittori, Cecilia Di Fabio, Andrea Scardaci, Francesco Smedile, Ilaria Mascilini, Elisa Francia, Corrado Cecchetti, Franco Marinangeli, Giuliano Marchetti, Teresa Grimaldi Capitello, Marco Cascella

**Affiliations:** 1 Department of Anesthesia, Critical Care and Pain Medicine, ARCO, Ospedale Pediatrico Bambino Gesù IRCCS, Piazza S. Onofrio 4, 00165 Rome, Italy; 2 Department of Life, Health and Environmental Sciences (MeSVA), University of L’Aquila, Piazzale Salvatore Tommasi 1, Blocco 11, Coppito, 67010 L’Aquila, Italy; 3 Department of Medical and Surgical Sciences, Anesthesia and Intensive Care, “Magna Graecia” University, Viale Europa, 88100 Catanzaro, Italy; 4 Surgery Unit, Bios Medical Center, Via Domenico Chelini 39, 00197 Rome, Italy; 5 Clinical Psychology Unit, Department of Neuroscience, Ospedale Pediatrico Bambino Gesù IRCCS, Piazza S. Onofrio 4, 00165 Rome, Italy; 6 Department of Medicine, Surgery and Dentistry, University of Salerno, Via Salvador Allende, 43, 84081 Baronissi, Italy

**Keywords:** metamizol, dipyrone, cannabinoid, children, pediatric anesthesia, postoperative pain, pediatric analgesia, opioid, NSAIDs, endocannabinoid system

## Abstract

**Background:** Metamizol (dipyrone) is a widely used analgesic and antipyretic drug in several European countries, particularly for postoperative pain management in both adult and pediatric populations. **Methods:** A narrative literature review was conducted to evaluate the efficacy, safety, and pharmacological mechanisms of metamizol in postoperative pain management. A comprehensive search of PubMed, Scopus, and the Cochrane Library was performed, and included articles published up to 2024. Search terms included *metamizol*, *dipyrone* and *children*. **Results:** The available evidence indicates that metamizol provides effective postoperative analgesia, with an efficacy comparable to that of other non-steroidal anti-inflammatory drugs and paracetamol. Pediatric studies similarly support its effectiveness in postoperative settings. Regarding safety, short-term use of metamizol appears to be well tolerated, with a low incidence of serious adverse events. Mechanistic studies suggest that metamizol exerts analgesic effects through a multimodal pathway, involving not only cyclo-oxygenase inhibition but also modulation of opioid and endocannabinoid systems. **Conclusions:** Metamizol represents an effective and generally well-tolerated option for short-term postoperative pain management in both adults and children when used under appropriate clinical monitoring. Current evidence supports a favorable benefit-to-risk balance for short-term use while highlighting the need for caution during prolonged therapy. Further large-scale, prospective studies are warranted to better define rare adverse events, clarify interindividual risk factors, and refine the understanding of their non-classical mechanisms of action.

## 1. Introduction

Metamizol is an analgesic, antipyretic, and spasmolytic drug belonging to the class of non-opioid analgesic, which are widely used in the treatment of acute postoperative pain, oncologic pain, colicky pain, and migraine [1]. Despite longstanding concerns related to rare but potentially severe adverse events, including agranulocytosis and anaphylactic reactions, metamizol remains one of the most commonly prescribed analgesics in several European countries, such as Germany, Austria, Switzerland, and Spain [2,3,4]. In contrast, its use has been banned in the United States, Great Britain, and Scandinavian countries [2,5,6,7].

**Figure 1:**
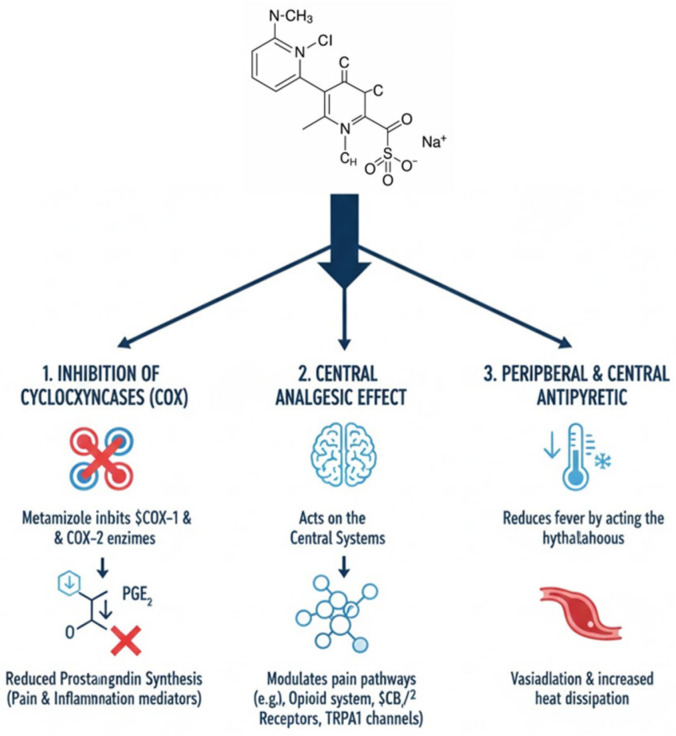
Mechanisms of metamizol (generated with assistance from Gemini Pro).

Robust evidence supports its analgesic efficacy. A Cochrane systematic review including 809 participants demonstrated that a single oral dose of metamizol 500 mg administered for acute postoperative pain achieved at least 50% pain relief in approximately 70% of patients, compared with about 30% receiving a placebo [8]. Metamizol is a prodrug that undergoes rapid hydrolysis to 4-methylaminoantipyrine (4-MAA), which represents the main active metabolite [9]. Increasing evidence suggests that its analgesic effect is not exclusively related to cyclo-oxygenase inhibition but also involves modulation of cannabinoid and opioid receptors [10]. In particular, the cannabinoid CB2 receptor, predominantly expressed in immune cells and keratinocytes, has been implicated in its antinociceptive mechanisms [11] (Figure 1).

Despite its widespread clinical use, safety concerns persist. Agranulocytosis, defined as a neutrophil count below 500/µL and potentially associated with pancytopenia, represents the most feared adverse event [12]. However, available data suggest that its incidence is low. In a multicenter study involving more than 1177 pediatric patients, adverse events related to metamizol occurred in fewer than 0.3% of cases, with no reported episodes of agranulocytosis [13]. Other reported adverse effects include chronic interstitial nephritis and gastrointestinal disturbances, as well as anaphylaxis, bronchospasm, and toxic epidermal necrolysis [14].

Metamizol therefore represents an important therapeutic solution in pediatrics. In fact, literature data shows that approximately 50% of pediatric hospitalized patients are not treated appropriately for pain [15]. This is due, on the one hand, to inadequate pain measurement and, on the other, to the use of drugs that undertreat pain [16]. One possible explanation is the unavailability of drugs approved for use in pediatrics [17,18]. Pediatric pharmacology research is severely limited due to sampling, safety, and ethical issues. This means that the medications available to pain specialists, and indeed to pediatricians in general, are limited and sometimes inadequate. Knowing how to properly utilize available resources and understanding the indications, limitations, and potential uses can make all the difference.

In this sense, metamizol may be a valuable aid for pain specialists as it can treat even moderate pain, including visceral pain.

## 2. Materials and Methods

A narrative literature review was conducted to evaluate the efficacy and safety of metamizol in both pediatric and adult populations. Clinical and observational studies were identified through systematic searches of PubMed, Scopus, and the Cochrane Library. Articles published up to 2024 were considered.

## 3. Results

The search strategy included the terms metamizol, dipyrone and children. Studies involving pediatric and/or adult patients treated with metamizol and reporting outcomes relevant to analgesic efficacy, postoperative use, or adverse events were eligible for inclusion. Articles not available in full text were excluded.

Overall, 112 publications were screened, and 58 studies were identified as relevant and included in the analysis. Owing to the substantial methodological heterogeneity among the included studies, a narrative synthesis was performed, including a descriptive evaluation of results and a critical comparison of the available literature.

## 4. Mechanism of Postoperative Pain

In patients undergoing surgical procedures, postoperative pain represents the most frequent symptom, resulting from a complex pathophysiological response to tissue injury [19]. The postoperative inflammatory phase is characterized by increased release of endogenous prostaglandins, particularly PGE_2_, which play a key role in nociceptor sensitization [20]. Experimental studies have demonstrated that metamizol inhibits carrageenan-induced hyperalgesia, supporting its analgesic effects in inflammatory pain models [21]. Using the same model, maximal hyperalgesia has been shown to peak approximately three hours after inflammatory stimulation [22,23].

The nociceptive stimulus also triggers central neuronal hyperexcitability, which progressively increases in the hours and days following surgery [24]. This central sensitization enhances synaptic pain transmission and contributes to the increased demand for analgesic medications during the early postoperative period. Postoperative pain in pediatric patients is well documented. An example of a painful intervention, among the most frequent in children, is tonsillectomy in which metamizol is indicated [25,26,27].

## 5. NSAIDs: Effects of Metamizol and Its Role in Postoperative Pain

Metamizol is a non-steroidal anti-inflammatory drug with analgesic and antipyretic properties primarily related to cyclo-oxygenase inhibition and subsequent reduction in prostaglandin synthesis [28]. Its analgesic activity has also been associated with modulation of the endogenous opioid system and inflammatory hyperalgesia pathways [29].

In five studies, an oral dose of 500 mg resulted in at least 50% pain relief in approximately 70% of treated patients compared with about 30% in the placebo group, with a reduced need for rescue analgesia over the following 4–6 h [8]. The role of metamizol in pediatric postoperative pain has been further supported by a 2021 systematic review including nine randomized clinical trials, which showed that both metamizol and paracetamol were superior to placebo, with no significant differences between the two agents [30].

In a prospective multicenter study involving 1177 children treated with metamizol in the postoperative period, the incidence of serious adverse events was below 0.3%, with no reported cases of agranulocytosis [13]. The incidence of agranulocytosis appears to vary considerably across countries, likely reflecting differences in study methodologies, population genetics, and the presence of polymorphic genes that remain incompletely characterized [30]. Comparative studies in adult surgical settings indicate that metamizol provides analgesic efficacy comparable to other NSAIDs or paracetamol. For instance, in patients undergoing breast surgery, intravenous metamizol demonstrated clinical equivalence to intravenous paracetamol for postoperative pain control [31].

## 6. Spasmolytic Effect

The pharmacological and experimental bases of metamizol’s spasmolytic activity remain heterogeneous and not completely clarified. Evidence shows that the antispastic effects of metamizol derive mainly from indirect mechanisms of neurogenic nature, rather than from a direct activity on smooth muscle. The spasmolytic activity seems to be associated with its action on ATP-dependent potassium channels and on cannabinoid receptors [6].

Metamizol probably reduces spasm of the sphincter of Oddi muscles by acting on beta2-adrenoceptors [32,33].

In vitro experimental studies on smooth muscle have reported controversial effects on the spasmolytic activity of metamizol [34]. This derives from the fact that metamizol is a pro-drug and may not exert a direct in vitro effect on the muscle; instead, it acts through its metabolites such as MAA [35]. However, when present, the spasmolytic effect of metamizol only appears at very high concentrations, as in the case of the guinea pig ileum, where metamizol at a dose of 10–100 mM showed no effect, whereas at a dose of 100–500 mM, the drug showed a myogenic spasmolytic effect for 30 min in the ileum [36].

The difference between experimental and clinical evidence becomes particularly evident when considering the effect of metamizol in the treatment of biliary colic. In humans, metamizol reduces the tone of the sphincter of Oddi [37], whereas the use of metamizol in animals did not modify biliary pressure nor the myoelectric activity of the sphincter of Oddi [38].

In the same clinical context, metamizol has proven more effective in controlling biliary colic pain than traditional spasmolytic drugs, reinforcing the idea that its efficacy derives from a combination of analgesic effects and visceral relaxation.

The activity of metamizol on intestinal colic remains doubtful as its effects seem to be mediated mainly by analgesic activity [32].

## 7. Metamizol: New Mechanisms for a Vintage Drug

In recent years, the analgesic mechanisms of metamizol that extend beyond prostaglandin inhibition have been increasingly recognized (Table 1). Although metamizol is generally considered to have weak anti-inflammatory activity, its mechanism of action is not completely clarified and probably involves additional non-traditional pathways [33]. The drug acts through a complex network of central and peripheral interactions. New metabolites capable of binding to cannabinoid receptors have been identified, suggesting the involvement of the endocannabinoid system [6].

This observation is consistent with experimental data according to which the dipyrone metabolite 4-methylaminoantipyrine acts as a CB1 agonist [39,40]. The direct connection between metabolites and CB1 receptors helps to understand why metamizol produces analgesia with a central component mediated by systems not typically involved with NSAIDs. The CB1 receptor is in fact involved in analgesia, catalepsy and hypolocomotion induced by the administration of metamizol. In addition to the endocannabinoid system, metamizol also appears to interact with the cyclooxygenase (COX) system in a different way compared with traditional NSAIDs. Metamizol reduces prostaglandin-induced hyperalgesia, an inflammatory mediator synthesized through activation of the COX enzyme [20]. Unlike classical COX inhibitors, prolonged treatment with metamizol does not induce COX-2 expression, does not modify COX-1 expression, and does not cause ulcers in the gastric mucosa [41]. The anti-inflammatory effect of the drug can also be explained by the mechanism through which metamizol inhibits COX. Metamizol and its metabolites MAA and AA, through redox mechanisms, sequester the radicals necessary for the catalytic activity of the enzyme, modifying its oxidative state [9]. This redox mechanism appears to underlie the modulation of TRPA1 and TRPV1 channels, which are activated and not inhibited by MAA and AA; this mechanism involves N-terminal cysteines, which are responsible for the activation of both channels [42,43,44]. Unlike classical NSAIDs, metamizol, although classified as a “non-opioid analgesic”, exerts weak anti-inflammatory and antinociceptive effects due to mechanisms different from simple peripheral COX inhibition [45]. Metamizol also acts on central systems, intervening on descending pain pathways. One of the targets is the PAG-RVM system [46]. The use of metamizol causes in rats an inhibition of the PAG through nociceptive circuits in the spinal cord, and this mechanism appears to be mediated by opioidergic mechanisms in the RVM [47].

After oral administration, metamizol is rapidly hydrolyzed to 4-MAA, which represents the main circulating metabolite and is responsible for most of its pharmacological interactions. Its half-life ranges between 2.6 and 3.5 h [48].

Another mechanism that may underlie pain reduction is inhibition of COX-3, a variant of COX-1 [49,50,51,52,53] that is expressed mainly in the central nervous system. In addition to COX inhibition, the opioid and cannabinoid systems also appear to be involved [48], particularly through cannabinoid-1 (CB1) receptors and vanilloid TRV1 receptors, which are expressed in the periaqueductal gray matter and the rostral ventromedial medulla [54]. In a recent study, it was demonstrated that the active metabolite of metamizol 4-methylaminoantipyrine exerts a partially local anti-allodynic effect that is partially dependent on CB2 receptors and kappa opioid receptors [55]. The endocannabinoid system is closely connected to the cyclooxygenase system, particularly COX-2. The endocannabinoids anandamide (AEA) and 2-arachidonoylglycerol (2-AG) derived from arachidonic acid are considered the main endocannabinoids [56,57]. In rats, administration of metamizol has no effect on 2-AG levels but causes a reduction in AEA levels in the RVM and spinal cord. Endocannabinoids are not only produced “on demand”, but are also rapidly degraded by specific enzymes [58]. AEA and 2-AG are removed from the extracellular space through a cellular uptake mechanism followed by enzymatic inactivation. AEA is mainly degraded into arachidonic acid and ethanolamine by fatty acid amide hydrolase (FAAH), while 2-AG is mainly metabolized into arachidonic acid and glycerol by monoacylglycerol lipase (MAGL) [59,60]. AEA and 2-AG represent not only substrates of FAAH and MAGL, but also substrates of other enzymatic systems, including those of cyclooxygenases, mainly COX-2 [61,62], as they produce prostamides and prostaglandin-glycerol esters. A reduction in these pro-inflammatory and pro-nociceptive mediators contributes to antinociceptive activity [61]. Metamizol increases the levels of anandamide and 2-AG by preventing their degradation by COX-2, contributing to the analgesic and antinociceptive effect of the drug [63]. COX inhibition significantly contributes to the increase in endocannabinoid tone because arachidonic acid mobilization increases AEA production [64]. To fully understand the activity of metamizol and its possible adverse events, it is also necessary to consider its role on the hepatic CYP450 system; it appears to be an inducer of CYP3A4, CYP2B6, and CYP2C19 enzymes [65]. Enzymatic activation, although not significant, also occurs after administration of a single dose of metamizol and reaches its maximal effect after 5 days of administration [66]. This phenomenon explains many clinical interactions observed with various types of drugs, immunosuppressants, sertraline, quetiapine, oral anticoagulants such as rivaroxaban, edoxaban, and antivirals [66]. This demonstrates that metamizol is not only an analgesic drug with complex mechanisms, but also a drug with an interaction profile with other substances that must be evaluated with extreme caution, especially in polypharmacological contexts.

**Table 1:** Non-classical mechanism of action of metamizol.

Non-Classical Mechanisms of Action of Metamizol		
Domain	Key Findings	Evidence [Ref]
Beyond COX inhibition	Analgesic effects not fully explained by prostaglandin suppression; involvement of central and peripheral pathways.	Experimental studies [6,25]
Cannabinoid system	Metamizol metabolites interact with cannabinoid receptors, contributing to central analgesia.	Experimental and in silico studies [6,40]
CB1 receptor activation	4-MAA acts as a CB1 activator to mediate central antinociceptive effects.	Experimental studies [40]
Descending pain modulation	Activation of PAG-RVM axis; involvement of opioid mechanism in RVM.	Animal studies [46,47]
COX redox modulation	Inhibition of COX activity via redox-dependent mechanisms without COX-2 induction or gastric injury.	Experimental studies [9,41]
TRP channels	Modulation of TRPA1 and TRPV1 channels through redox-sensitive cysteine residues.	Experimental studies [42,43,44]
Peripheral antinociception	Local antihyperalgesic effect mediated by CB2 and k-opioid receptors.	Experimental studies [55]
Endocannabinoid tone	Indirect modulation of endocannabinoid signaling via COX inhibition and altered anandamide metabolism.	Experimental studies [59,61,62,63,64]

## 8. The Endocannabinoid System in Chronic Pain

The endocannabinoid system is one of the main modulators of chronic pain due to its ability to influence both central sensitization and neuroinflammation (Table 2). CB1 and CB2 receptors constitute the core of the endocannabinoid system. CB1 is predominantly expressed in brain structures and peripheral tissues, whereas CB2 is expressed in cells of the immune system at the peripheral level. Most of the adverse effects associated with cannabinoid receptor agonists are mediated by CB1 receptors [61,67]. This distribution highlights that the endocannabinoid system is simultaneously involved in nociceptive transmission and in the regulation of inflammatory responses. Activation of the endocannabinoid system is associated with a limitation of neuroinflammation [68,69]. Particularly important is the activation of CB2 receptors located in immune cells and peripheral nerves, which are involved in reducing inflammation and hyperalgesia [70,71]. This activation is essential to counteract the persistence of painful stimuli since neuroinflammation is one of the main causes of the transition from acute pain to chronic pain. Activation of CB1 receptors in the latero-ventrolateral PAG and in the RVM modulates descending inhibitory pain pathways [72,73]. The antinociceptive activity associated with CB2 receptor activation provides antinociceptive effects through the opioid system present on the terminals of primary afferent neurons [74], including peripheral ones [75,76]. The endocannabinoid system participates in pain modulation through different mechanisms. CB1 receptors are distributed in particular at the level of the cerebellum, hippocampus, basal ganglia, amygdala, spinal cord, and cerebral cortex [59]. This distribution allows CB1 receptors to regulate neuronal excitability and the release of major cannabinoid neurotransmitters. CB1 activation stimulates circuits that suppress the release of neurotransmitters like GABA and diminishes the release of neurotransmitters such as glutamate [77]. Recent studies have underscored the increasing significance of the CB2 receptor in the pathophysiology of pain, particularly regarding neuroimmune mechanisms. This immunomodulatory function is essential for the regulation of neuroinflammation, despite the initial perception of the CB2 receptor as being primarily associated with immune responses [78]. The activation of CB2 receptors is correlated with the inhibition of pro-inflammatory cytokines, including IL-1β and TNF-α [79,80], a phenomenon central to preventing peripheral and central sensitization in chronic pain. Other endocannabinoids such as anandamide and 2-AG contribute to the regulation of nociception as retrograde messengers capable of modulating synaptic activity. These messengers participate in the regulation of nociceptive signals through interaction with CB1 and CB2 [81]; in particular, anandamide exerts its action by binding to CB1 receptors, whereas 2-AG contributes to pain modulation under conditions of stress or tissue injury [81].

In chronic pain, an increase in circulating endocannabinoid levels is frequently observed; for example, 2-AG levels are increased in chronic pain conditions [82,83]. Prolonged activation of the endocannabinoid system over time is associated with the development of allodynia; indeed, it has been documented that sustained production of endocannabinoids in response to nociceptive signals renders spinal neurons more reactive to non-painful stimuli [84]. Microglia represent a key element in the modulation of chronic pain by the endocannabinoid system; in the presence of persistent nociceptive stimulation, microglial cells undergo marked activation [85,86,87] which is accompanied by morphological and functional changes, leading to the establishment of central sensitization processes.

Microglial activation present in chronic pain is sustained by a series of signals originating from primary nociceptive neurons, which induce a rapid proliferation of microglia themselves. These cells acquire a functional profile of a pro-inflammatory type, corresponding to the M1 phenotype [88]. In this condition, microglia become a source of pro-inflammatory mediators such as IL-1β, TNF-α, IL-6, and PGE_2_, which act directly on spinal neurons, leading to central sensitization [89]. The resolution of the inflammatory response necessitates a functional reorganization of microglia towards an M2 phenotype, characterized by the ability to secrete mediators such as IL-10, TGF-β, IL-4, and IL-13, which exhibits phagocytic activity with a predominantly anti-inflammatory effect [90].

In this context, the CB2 receptor assumes particular importance, because its expression is markedly increased in activated microglia [91,92] and it is able to attenuate pain transmission through the control of microglial activity and the suppression of neuroinflammatory processes [93]. CB2-mediated signaling is associated with a functional shift of microglia from a pro-inflammatory M1 phenotype to an M2 phenotype with neuroprotective characteristics; this involves not only structural modifications, but also a functional recalibration of microglia, with an increase in the production of anti-inflammatory cytokines and the production of M2-associated factors, which are associated with a reduction in neuronal hyperexcitability linked to central sensitization processes [94]. This mechanism is essential in controlling the propagation of pain circuits and in preventing the stabilization of chronic pain [91,92,93,94]. The endocannabinoid system plays a key role in this change by changing how microglia express the CB2 receptor, which helps to reduce nociceptive signaling cascades [95]. On the other hand, CB1 receptor expression is very low, as shown by the fact that Cnr1 transcripts are not found in microglia that are not activated [96].

**Table 2:** Endocannabinoid system in chronic pain.

Endocannabinoid System in Chronic Pain		
Domain	Key Findings	Evidence [Ref]
Pain modulation	Regulates nociception, sensitization and neuroinflammation.	Reviews [56,59]
CB1 receptors	Central modulation of neuronal excitability and descending inhibition.	Experimental studies [59,72]
CB2 receptors	Peripheral and immune-mediated analgesia.	Experimental studies [81]
Opioid interaction	CB2 activation induces peripheral opioid-mediated analgesia.	Experimental studies [74,75,76]
Endocannabinoids	Anandamide and 2-AG act as retrograde modulators of nociceptive transmission.	Experimental studies [81]
Chronic pain state	Increased endocannabinoid levels associated with persistent pain.	Clinical studies [82,83]
Microglia	Microglial activation sustains central sensitization.	Experimental studies [85,87,91]
CB2-microglia axis	CB2 signaling limits neuroinflammation and promotes M2 phenotype.	Experimental studies [91,92,93,94,95]

## 9. Efficacy and Safety in Clinical Practice

The efficacy and safety of metamizol in clinical practice are supported by data from randomized controlled trials and observational studies. A 2015 meta-analysis including 79 randomized trials found no significant differences in overall adverse events between metamizol and placebo, paracetamol, aspirin, or other NSAIDs. Notably, metamizol was associated with a lower incidence of neurological adverse effects, such as vertigo, dizziness, and headache (15 vs. 57 events; RR = 0.50, 95% CI 0.27–0.92) [2]. Improved gastrointestinal tolerability was also observed, with vomiting reported in 12 metamizol-treated patients compared with 54 patients receiving opioids (RR = 0.48) [2].

In most included trials, treatment duration was limited, supporting the observation that adverse events associated with metamizol are closely related to treatment length. A large prospective study reported that metamizol was administered in 31.7% of inpatient stays, predominantly via parenteral routes. During follow-up, three cases of agranulocytosis, one case of allergic shock, and one case of rash were reported, all occurring after prolonged administration and resolving without permanent sequelae. The risk of agranulocytosis typically manifested after approximately 28 days of continuous treatment [97].

Overall, the available evidence supports the effectiveness of metamizol in postoperative pain management, with fewer neurological and gastrointestinal adverse effects than opioids and a low incidence of severe complications when used short-term in hospital settings. Nevertheless, caution is warranted with prolonged therapy due to the potential for delayed hematological toxicity (Table 3).

## 10. Conclusions and Future Perspectives

Metamizol represents an effective analgesic option for pediatric patients in the postoperative setting, with a favorable tolerability profile when used short term under clinical monitoring. Evidence indicates that metamizol provides analgesic efficacy comparable to weak opioids while being associated with fewer neurological and gastrointestinal adverse effects [101]. However, prolonged use may result in severe hematological complications, particularly agranulocytosis, which typically occurs several weeks after treatment initiation.

Accordingly, the current evidence supports restricting metamizol use to short-term interventions, with heightened vigilance during prolonged therapy or in patients with potential hematological vulnerability. From a pharmacological perspective, emerging data highlight mechanisms of action extending beyond COX inhibition, including interactions with the endocannabinoid system and intracellular signaling pathways involved in pain modulation. These findings open new research avenues aimed at clarifying the contribution of active metabolites to clinical analgesia and identifying genetic determinants underlying interindividual variability in efficacy and adverse events.

Although there is robust literature in the pediatric field regarding the use of metamizol, in some respects, it is necessary to adopt the evidence obtained from studies on the adult population, as is necessary in the field of pediatric algology [102].

All these data indicate that it is an ideal drug for perioperative use in pediatric patients. It has two major and important indications. The first indication is for its use in the postoperative period in pediatric day surgery. There is now a robust body of literature demonstrating that the primary cause of unplanned admissions following pediatric day surgery is pain [103]. Indeed, what should be a specific activity of an acute pain service is now delegated to the parents of children undergoing surgery to perform [104,105]. This drug has been proven to be as effective as opioids in some cases, without the social stigma or prejudice that unfortunately burden this class of drugs, making metamizol a valid tool for treating postoperative pain in day surgery [101]. It should also be emphasized that the most feared adverse reaction associated with metamizol—agranulocytosis—is linked to prolonged use. Therefore, short-term use can be considered safe, with a risk/benefit ratio that favors the benefits. As we have seen, this drug can treat moderate pain, even visceral pain, and is characterized by rapid action. It should not be overlooked that the critical period for the onset of chronic post-surgical pain is precisely the very early stages, such as when systemic therapy is combined with regional anesthesia. Therefore, even in this case, prolonged—and excessive—use, which could put patients at risk, is avoided. Furthermore, metamizol’s cannabinoid activity may play a protective role in preventing the onset of chronic pain, which is multifactorial. And here the second indication emerges: the prevention of chronic post-surgical pain.

The use of multimodal analgesia to manage perioperative pain is now well established, both in terms of scientific evidence and clinical practice, in order to minimize side effects and maximize patient comfort [106,107]. Despite their limitations, NSAIDs still play an important role in managing acute postoperative pain, especially in settings such as day surgery. Using a drug that not only has a broad spectrum of actions, all potentially useful for pain management, represents a significant added value for pain management.

The chronicization of acute postoperative pain has significant social and economic implications. Poorly managed pain translates into prolonged hospitalizations, representing a direct cost in terms of bed occupancy and increased healthcare costs. However, a patient with inadequate pain control is at significant risk of developing chronic pain [108,109]. This leads to increased indirect costs, especially in pediatrics. A child unable to lead a normal social life due to pain represents not only a healthcare priority, but also a burden on the entire family. The time required for pediatric patients’ rehabilitation translates into lost workdays for parents/guardians, who in turn, as caregivers, risk becoming ill. It is therefore clear that investments, both in healthcare and research, aimed at combating chronic pain translate into a winning choice in the medium to long term, resulting in guaranteed socioeconomic savings.

Future research should prioritize large, multicenter prospective studies to better define the true incidence of rare adverse events, identify specific risk factors, and characterize pharmacokinetic profiles across diverse patient populations, particularly in pediatrics. Further exploration of novel mechanisms may ultimately support the development of safer and more selective analgesic strategies, optimizing the role of metamizol in contemporary clinical practice.

## Figures and Tables

**Table 3:** Evidence from efficacy, safety, and mechanisms of action of metamizol.

Domain	Key Findings	Evidence [Ref]
**Analgesic efficacy**	Effective in acute postoperative pain, oncologic pain, colicky pain, and migraine. A single oral dose of 500 mg provides ≥50% pain relief in ~70% of patients.	Cochrane reviews and RCTs [8]
**Postoperative pain (adults)**	Comparable analgesic efficacy to other NSAIDs and paracetamol; reduced need for rescue analgesia within 4–6 h.	Systematic reviews, comparative trials [8,31]
**Postoperative pain (pediatrics)**	Effective for postoperative pain control; similar efficacy to paracetamol and superior to placebo.	Systematic review, observational studies [13,30]
**Mechanism—classical**	Inhibition of cyclo-oxygenases with reduced prostaglandin synthesis (PGE_2_) leading to decreased peripheral sensitization.	Experimental and pharmacological studies [20,28]
**Mechanism—non-classical**	Activation/modulation of opioid and cannabinoid systems; involvement of CB1 and CB2 receptors; modulation of endocannabinoid tone.	Experimental studies [6,10,98,99,100]
**Active metabolites**	Prodrug rapidly hydrolyzed to 4-methylaminoantipyrine (4-MAA); further metabolized into active derivatives with central and peripheral analgesic effects.	PK/PD and experimental data [9,10,46]
**Safety—common adverse events**	Generally well tolerated; lower incidence of neurological and gastrointestinal adverse effects compared with opioids.	Meta-analyses, RCTs [2]
**Safety—serious adverse events**	Agranulocytosis is rare, typically associated with prolonged use (>2–4 weeks); very low incidence in short-term postoperative use.	Observational and pharmacovigilance studies [7,12,13,97]
**Clinical considerations**	Suitable for short-term postoperative analgesia under monitoring; caution required for prolonged therapy and in patients at hematological risk.	Guidelines and expert recommendations [12]

## Data Availability

No new data were created or analyzed in this study.
